# R7-binding protein targets the G protein β5/R7-regulator of G protein signaling complex to lipid rafts in neuronal cells and brain

**DOI:** 10.1186/1471-2091-8-18

**Published:** 2007-09-19

**Authors:** Lylia Nini, Abdul A Waheed, Leelamma M Panicker, Meggan Czapiga, Jian-Hua Zhang, William F Simonds

**Affiliations:** 1 Metabolic Diseases Branch, 10/8C-101, National Institute of Diabetes and Digestive and Kidney Diseases, National Institutes of Health, Bethesda, MD 20892, USA; 2 Virus-Cell Interaction Section, HIV Drug Resistance Program, National Cancer Institute at Frederick, Frederick, MD 21702, USA; 3 Research Technologies Branch, Bldg. 4Room B2-30B, National Institute of Allergy and Infectious Diseases, National Institutes of Health, Bethesda, MD 20892, USA

## Abstract

**Background:**

Heterotrimeric guanine nucleotide-binding regulatory proteins (G proteins), composed of Gα, Gβ, and Gγ subunits, are positioned at the inner face of the plasma membrane and relay signals from activated G protein-coupled cell surface receptors to various signaling pathways. Gβ5 is the most structurally divergent Gβ isoform and forms tight heterodimers with regulator of G protein signalling (RGS) proteins of the R7 subfamily (R7-RGS). The subcellular localization of Gβ 5/R7-RGS protein complexes is regulated by the palmitoylation status of the associated R7-binding protein (R7BP), a recently discovered SNARE-like protein. We investigate here whether R7BP controls the targeting of Gβ5/R7-RGS complexes to lipid rafts, cholesterol-rich membrane microdomains where conventional heterotrimeric G proteins and some effector proteins are concentrated in neurons and brain.

**Results:**

We show that endogenous Gβ5/R7-RGS/R7BP protein complexes are present in native neuron-like PC12 cells and that a fraction is targeted to low-density, detergent-resistant membrane lipid rafts. The buoyant density of endogenous raft-associated Gβ5/R7-RGS protein complexes in PC12 cells was similar to that of lipid rafts containing the palmitoylated marker proteins PSD-95 and LAT, but distinct from that of the membrane microdomain where flotillin was localized. Overexpression of wild-type R7BP, but not its palmitoylation-deficient mutant, greatly enriched the fraction of endogenous Gβ5/R7-RGS protein complexes in the lipid rafts. In HEK-293 cells the palmitoylation status of R7BP also regulated the lipid raft targeting of co-expressed Gβ5/R7-RGS/R7BP proteins. A fraction of endogenous Gβ5/R7-RGS/R7BP complexes was also present in lipid rafts in mouse brain.

**Conclusion:**

A fraction of Gβ5/R7-RGS/R7BP protein complexes is targeted to low-density, detergent-resistant membrane lipid rafts in PC12 cells and brain. In cultured cells, the palmitoylation status of R7BP regulated the lipid raft targeting of endogenous or co-expressed Gβ5/R7-RGS proteins. Taken together with recent evidence that the kinetic effects of the Gβ5 complex on GPCR signaling are greatly enhanced by R7BP palmitoylation through a membrane-anchoring mechanism, our data suggest the targeting of the Gβ5/R7-RGS/R7BP complex to lipid rafts in neurons and brain, where G proteins and their effectors are concentrated, may be central to the G protein regulatory function of the complex.

## Background

Seven transmembrane-spanning receptors in eukaryotes regulate intracellular processes in response to extracellular signals through their interaction with signal-transducing heterotrimeric guanine-nucleotide binding regulatory proteins (G proteins^1^) [1]. cDNAs from five G protein β subunit genes (Gβ1–5) have been identified by molecular cloning. The Gβ5 isoform shares much less homology with other isoforms (~50%) and is preferentially expressed in brain [2]. A longer splice variant of Gβ5, Gβ5L, is present in retina [3]. Gβ5 and Gβ5L, but not the other Gβ isoforms, can form tight heterodimers with the R7 subfamily of regulator of G protein signaling (RGS) proteins: RGS6, 7, 9 and 11 (R7-RGS) [4-10], an interaction mediated by a Gγ-like (GGL) domain present in the R7 subfamily of RGS proteins [5,7].

The function of Gβ5/R7-RGS protein complexes in brain and the role of the subcellular localization of the complex in such function are unclear. We previously demonstrated the multi-compartmental subcellular localization of Gβ5 and R7 proteins to the plasma membrane, cytosol, and cell nucleus in neurons and brain using subcellular fractionation and confocal microscopy [11]. We also found that the interaction of Gβ5 with the GGL-domain containing RGS proteins directs its nuclear localization [12]. Previous work with recombinant Gβ5-RGS7 complex expressed in Sf9 insect cells suggested it was the palmitoylation status of RGS7 that determined the membrane versus cytosolic localization of the complex [13]. More recently an R7 binding protein (R7BP) was discovered that binds tightly to Gβ5-R7 protein complexes [14,15]. R7BP is itself palmitoylated and can regulate the nuclear localization of the Gβ5/R7-RGS/R7BP protein complex based on the palmitoylation status of R7BP [15,16]. In its palmitoylated form, R7BP anchors Gβ5 protein complexes to the plasma membrane, and depalmitoylation of R7BP promotes translocation of Gβ5/R7-RGS/R7BP complexes to the nucleus via a polybasic nuclear localization signal (NLS) present near the C-terminus of R7BP [15-17]. These data invite further investigation into the nature of the membrane localization of Gβ5/R7-RGS/R7BP complexes.

To gain further insight into the effects of R7BP palmitoylation on the membrane targeting of Gβ5/R7-RGS/R7BP complexes we studied wild-type R7BP and the palmitoylation-deficient R7BP mutant proteins in transfected PC12 and HEK-293 cells. PC12 cells have neuron-like features including the ability to synthesize dopamine and norepinephrine and to express receptors for nerve growth factor, while HEK-293 have a non-neuronal phenotype. We report that Gβ5/R7-RGS/R7BP complexes localize to lipid raft microdomains in membranes from both cell types and in adult mouse brain and that the palmitoylation status of R7BP appears to control such lipid raft association. Together with recent evidence showing the regulatory effects of the Gβ5 complex on GPCR signaling are greatly enhanced by R7BP palmitoylation and membrane anchoring [17], our data suggests the targeting of the Gβ5/R7-RGS/R7BP complex to lipid rafts in neuronal cells may be critical for the G protein-directed function of the complex.

## Results

### Localization of endogenous Gβ5 and RGS7 to lipid raft membrane domains in native PC12 cells requires palmitoylation

The recently discovered R7BP is a SNARE-like protein with twin C-terminal cysteine residues that are covalently modified by palmitoylation [14,15]. R7BP binds tightly to Gβ5/R7-RGS protein complexes and can regulate the subcellular distribution of the Gβ5/R7-RGS/R7BP protein complex based on its palmitoylation status [15,16]. The covalent fatty acylation of several signalling proteins with palmitate results promotes their targeting to lipid rafts, plasma membrane microdomains enriched in cholesterol and glycosphingolipids (reviewed in [18,19]). Such proteins include H-Ras [20] and certain dually acylated heterotrimeric G protein α subunits [21].

**Figure 1:**
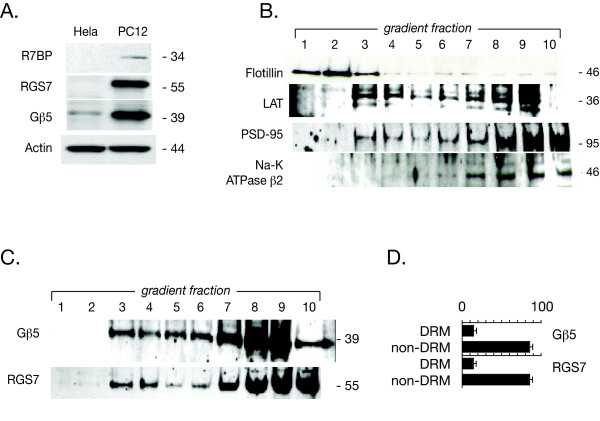
**Localization of endogenous Gβ5/RGS7 complexes to lipid rafts in PC12 cells**. A. Gβ5/RGS7/R7BP proteins are expressed endogenously in PC12 cells. Hela and PC12 cells were lysed and samples were loaded in SDS-PAGE gels and subjected to immunoblotting using anti-R7BP (N-terminal), anti-RGS7, anti-Gβ5, and actin antibodies as described in Methods. The relative mobility of the major immunoreactive bands (in kDa) is indicated on the right. B. Determination of DRM (lipid raft) and non-DRM markers along a sucrose density gradient in PC12 cells. The DRM (lipid rafts) were isolated using a sucrose density gradient prepared by ultracentrifugation as detailed in Methods. Fractions were analyzed by immunoblotting for flotillin-1, LAT and PSD-95 as markers for DRM and the β2 subunit of Na-K ATPase as a non-DRM marker. Fractions 1–5 were enriched in the DRM marker, while fractions 6–10 carried the non-DRM marker. C. Localization of endogenous Gβ5 and RGS7 proteins in PC12 cells. PC12 cells were lysed, and subjected to a sucrose density gradient. Equal aliquots from all ten fractions were subjected to SDS-PAGE followed by immunoblotting for the endogenous Gβ5 and RGS7 proteins as indicated. D. Densitometric quantification of the distribution of endogenous Gβ5 and RGS7 proteins among DRM and non-DRM fractions in sucrose density gradients. Immunoblots of sucrose density gradients such as that shown in C were analyzed by densitometry and the distribution of the indicated immunoreactivity between DRM and non-DRM fractions (as % of total immunoreactivity with that antibody) is shown as a histogram to the right of the corresponding immunoblots, with error bars representing the S.E.M. The results shown are representative of three experiments completed with similar results.

The dual palmitoylation of R7BP would make it a candidate for lipid raft targeting and we therefore looked for evidence of such targeting by established biochemical methods using neuron-like PC12 cells as an initial model system. Protein subunits encoding the Gβ5/R7-RGS/R7BP complex components are expressed in PC12 cells but not in non-neuronal HeLa (Fig. 1A) or HEK-293 cells (not shown) consistent with previous results [11]. In order to isolate lipid rafts we analyzed Triton-X-100 solubilizates on discontinuous sucrose density gradients by ultracentrifugation. The low buoyant density of detergent-resistant membranes (DRMs), which are aggregates of lipid rafts [22], causes them to float towards the top of sucrose gradients while detergent-soluble proteins are distributed toward the bottom. In the discontinuous sucrose density gradient methodology employed here for cultured cells, we used established lipid raft markers including flotillin-1 and linker for activation of T cells (LAT) (Fig. 1B) [23,24]. Another positive control for lipid raft isolation was the PSD-95 protein (Fig. 1B). PSD-95 is found in the brain localized to both synaptic lipid rafts and postsynaptic densities [25,26], membrane compartments between which certain protein components may communicate [27].

Using the discontinuous sucrose density gradient analysis, we estimate that 10 to 20% of endogenous Gβ5 and RGS7 proteins in naïve PC12 cells are localized to the lipid raft fractions (Fig. 1C and 1D). The low-density peak of endogenous RGS7 and Gβ5 proteins comigrates with the LAT and PSD-95 marker peaks in fractions 3 and 4 of the discontinuous sucrose gradient, comigrating also with the flotillin in fraction 3 just behind the peak of the flotillin marker in fraction 2. The bulk of the Gβ5/RGS7 proteins is found in the heavier sucrose fractions however, and comigrates with the Na-K ATPase β2 subunit marker used as a non-lipid raft negative control (Fig. 1B, C, D). Overnight treatment of PC12 cells with 2-bromopalmitate to block palmitoylation of endogenous proteins prevented targeting of the native Gβ5/RGS7 complex to the raft fractions, instead targeting the endogenous complex to the cell nucleus (not included in the gradient fractions) (data not shown), as previously demonstrated for transfected Gβ5/RGS9-1/GFP-R7BP complexes [15,17]. Taken together these results indicate that approximately 10 to 20% of endogenous Gβ5/RGS7 protein complexes in naïve PC12 cells are localized to a type of lipid raft with buoyant density similar to the lipid rafts containing the palmitoylated marker proteins PSD-95 and LAT, but distinct from the membrane microdomain where flotillin is localized. The localization of the Gβ5/RGS7 protein complex to the lipid raft fraction may require the palmitoylation of one or more endogenous proteins such as R7BP. In these experiments, the detection of endogenous R7BP in the sucrose gradient fractions from PC12 cells was not possible with the currently available antibodies due to the low levels of protein when diluted across the gradient.

### The palmitoylation status of R7BP governs partitioning of the Gβ5/R7-RGS complex to a Triton-insoluble fraction in PC12 cells

**Figure 2:**
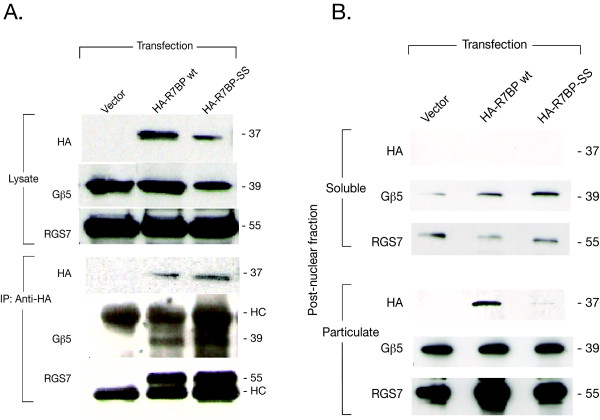
**Expression and interaction with endogenous Gβ5/RGS7 proteins of wild-type and mutant R7BP in PC12 cells**. A. Co-immunoprecipitation of endogenous Gβ5/RGS7 with transfected R7BP constructs in PC12 cells. Cells were transfected with pcDNA3 (vector), wild type (wt) HA-R7BP, or the non-palmitoylated HA-R7BP-SS mutant cDNAs. The detergent lysate was immunoprecipitated using anti-HA antibody. The whole cell lysate (upper) and the precipitated proteins (lower) were analyzed by immunoblotting using anti-HA, anti-RGS7, and anti-Gβ5 (ATDG) antibodies as shown. The relative mobility of the major immunoreactive bands (in kDa) and the immunoglobulin heavy chain (HC) are indicated on the right. B. Fractionation analysis of PC12 cells transfected with wild type R7BP or its non-palmitoylated mutant. PC12 cells transfected with pcDNA3 (vector), wild type (wt) HA-R7BP, or the non-palmitoylated HA-R7BP-SS mutant were lysed, and after a low-speed spin to remove unbroken cells and nuclei, were separated into post-nuclear soluble fraction (cytosol) and particulate fractions (crude membranes) as shown, by ultracentrifugation. Localization of transfected HA-R7BP, and endogenous Gβ5 and RGS7 were detected by immunoblotting in both soluble and particulate fractions using appropriate antibody as indicated in Methods. These experiments were performed twice with similar results, as shown here.

**Figure 3:**
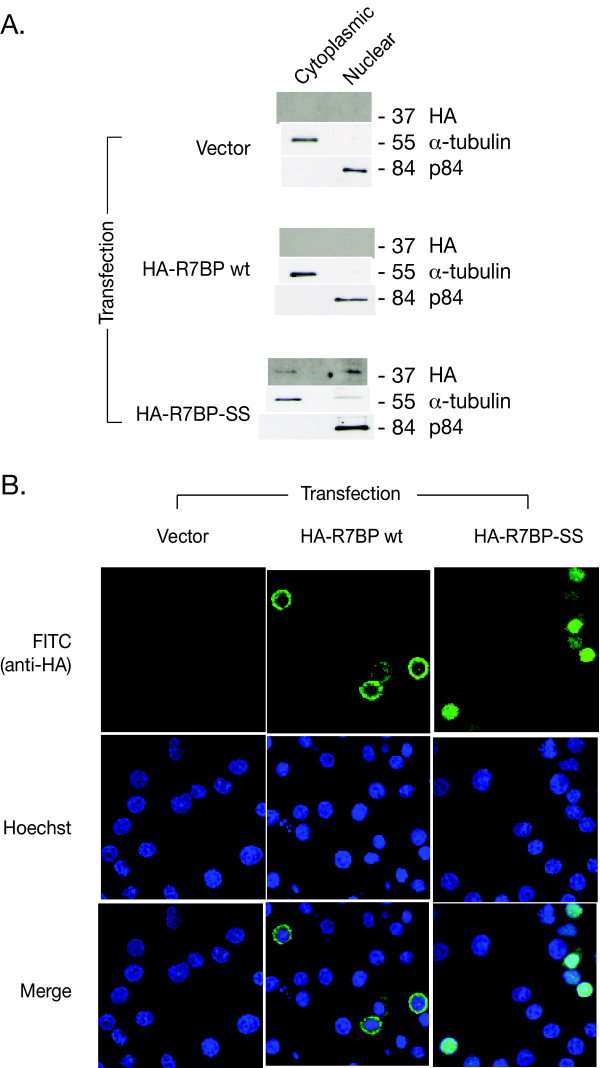
**Subcellular distribution of wild-type and mutant R7BP in PC12 cells**. A. Cytoplasmic and nuclear distribution of transfected wild type R7BP or non-palmitoylated mutant R7BP in PC12 cells. Cytoplasmic and nuclear fractions were prepared from PC12 cells transfected with the cDNAs indicated on the left. Immunoblots to monitor HA-R7BP expression, α-tubulin expression as a marker for the cytosolic fraction, and p84/N5 expression as marker for the nuclear fraction are shown. B. Immunocytofluorescent localization of R7BP in PC12 cells. PC12 cells transfected with vector (panels in left column), wild type (wt) HA-R7BP (middle column) or HA-R7BP SS (right column), were subjected to the immunostaining using anti-HA primary antibody and fluorescein isothiocyanate (FITC)-conjugated donkey anti-mouse IgG antibody (green signal) as a secondary antibody. The Hoechst 33342 nuclear dye (blue signal) was used to counterstain transfected PC12 cells. The single or combined (merge) fluorescent signal was recorded by laser confocal microscopy. The results shown are representative of three total experiments performed with similar findings.

The above results suggest that palmitoylation is required for the lipid raft targeting of native Gβ5/R7-RGS protein complexes but do not identify which palmitoylated protein(s) may be necessary for such targeting. We first investigated the role of the palmitoylated anchor protein R7BP in targeting Gβ5/R7-RGS protein complex to DRMs. To this end, we increased the expression level of R7BP by overexpressing either wild-type or mutant R7BP and checked their ability to interact with endogenous Gβ5/R7-RGS protein complexes and their subcellular localization. Both wild-type R7BP and the previously described palmitoylation-deficient R7BP- C252S/C253S double mutant (R7BP-SS) were readily expressed in PC12 cells in N-terminal HA epitope tagged form (Fig. 2A, upper) [15]. Immunoprecipitation of the transfected epitope-tagged R7BP constructs demonstrated their tight binding to, and co-immunoprecipitation with, endogenous Gβ5 and RGS7 (Fig. 2A, lower), consistent with previous reports employing non-palmitoylated R7BP mutants [14,15]. Subcellular fractionation of PC12 cells transfected with R7BP or R7BP-SS demonstrated differential localization of the wild-type and mutant proteins. Wild-type R7BP localized to the post-nuclear crude membrane fraction (Fig. 2B, center column), while the R7BP-SS mutant localized predominantly to the cytosolic and nuclear fractions (Fig. 2B, right-hand column; Fig. 3A, bottom panels). In order to better define the subcellular targeting of transfected R7BP and R7BP-SS in PC12 cells, we further analyzed their expression by laser confocal microscopy (Fig. 3B). The wild-type R7BP expressed in transfected PC12 cells excluded the cell nuclei that were directly identified by counterstaining with Hoechst reagent (Fig. 3B, middle column), consistent with the localization of endogenous R7BP in NG108-15 cells [16]. In contrast, the palmitoylation-deficient R7BP-SS mutant localized throughout the cells including the entire PC12 cell nucleus (Fig. 3B, right hand column). These findings are consistent with the previously described subcellular localization of wild-type and non-palmitoylated R7BP mutant proteins expressed in cultured cells and neurons [15,16].

**Figure 4:**
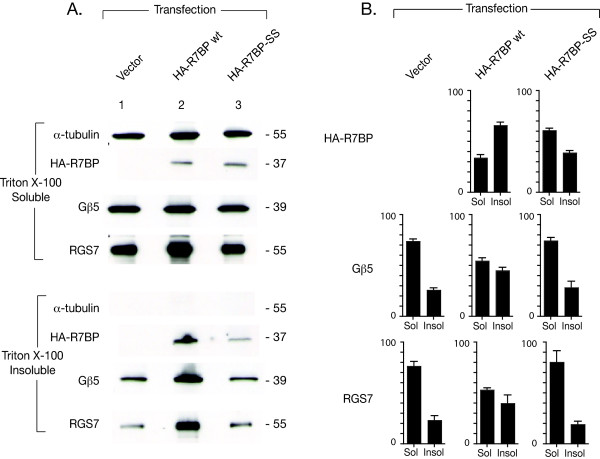
**Effect of R7BP on the distribution of endogenous Gβ5/RGS7 proteins into Triton X-100 soluble and insoluble fractions in PC12 cells**. A. PC12 cells transfected with vector only (left), wild type (wt) HA-R7BP (middle) or HA-R7BP SS (right) were harvested, lysed and fractionated into Triton X-100 soluble and insoluble fractions. Proteins in each fraction were probed for HA-R7BP, Gβ5 and RGS7 and α-tubulin by immunoblotting with specific antibodies as shown. The data shown are representative of three experiments performed with similar results. B. The percentage of total transfected HA-R7BP or endogenous Gβ5 or RGS7 protein in the Triton X-100 soluble (Sol) or insoluble (Insol) fractions was determined by densitometry for the different transfection conditions as shown, with error bars representing the S.E.M.

Because the insolubility of dually palmitoylated lipid raft-associated proteins in ice-cold Triton X-100 has been used previously in PC12 cells to define and isolate lipid rafts [28], we tested whether transfected R7BP could influence the distribution of endogenous Gβ5/R7-RGS proteins to these membrane microdomains. PC12 cells transfected with vector alone, HA-R7BP, or HA-R7BP-SS were extracted with cold Triton X-100 and separated into soluble and insoluble fractions (Fig. 4A). Immunoblotting showed that the transfected wild-type R7BP was found largely in the insoluble fraction while most of the R7BP-SS was soluble (Fig. 4A, cf. lanes 2 and 3). Furthermore while the bulk of the endogenous PC12 Gβ5 and RGS7 proteins were in the soluble fraction in the control and R7BP-SS transfected cells, some 40% of the endogenous Gβ5 and RGS7 shifted to the Triton-insoluble fraction when wild-type R7BP was transfected (Fig. 4A lane 2, cf. lanes 1 and 3; Fig. 4B). No shift to the Triton-insoluble fraction of α-tubulin, which is not a component of Gβ5/R7-RGS protein complexes with R7BP, was observed (Fig. 4A).

### R7BP regulates the targeting of Gβ5 and RGS7 to lipid raft membrane domains

**Figure 5:**
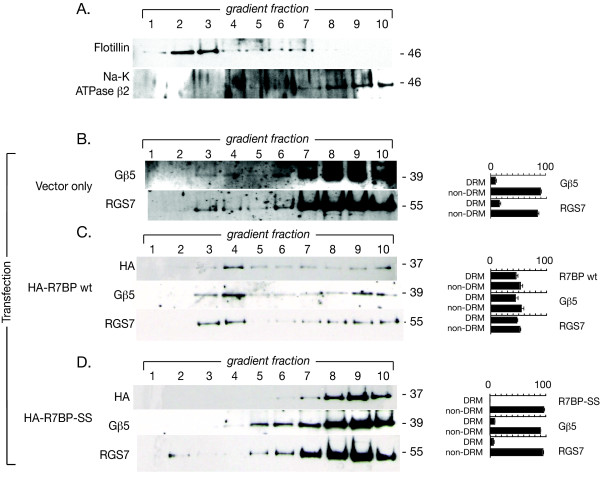
**Effect of R7BP on the distribution of the endogenous Gβ5/RGS7 complex among detergent-resistant membrane (DRM) and non-DRM fractions in PC12 cells**. A. Determination of DRM (lipid raft) and non-DRM markers along a sucrose density gradient in vector-transfected PC12 cells. The DRM (lipid rafts) were isolated using a sucrose density gradient prepared by ultracentrifugation as detailed in Methods. Fractions were analyzed by immunoblotting for flotillin-1 as a marker for DRM and the β2 subunit of Na-K ATPase as a non-DRM marker. Fractions 1–5 were enriched in the DRM marker, while fractions 6–10 carried the non-DRM marker. B. Localization of endogenous Gβ5 and RGS7 proteins in control PC12 cells transfected with empty pcDNA3-HA vector (vector only). PC12 cells were transfected with empty vector, lysed, and subjected to a sucrose density gradient centrifugation. Equal aliquots from all ten fractions were subjected to SDS-PAGE followed by immunoblotting for the endogenous Gβ5 and RGS7 proteins as indicated. Immunoblots of the sucrose density gradients shown were analyzed by densitometry and the distribution of the indicated immunoreactive bands between DRM and non-DRM fractions (as % of total immunoreactivity with that antibody) is shown as a histogram to the right of the corresponding immunoblot, with error bars representing the S.E.M. C. Localization of endogenous Gβ5 and RGS7 proteins in PC12 cells expressing wild type (wt) HA-R7BP. PC12 cells were transfected with wt HA-R7BP and analyzed as indicated in the legend to B. D. Localization of endogenous Gβ5 and RGS7 proteins in PC12 cells expressing the non-palmitoylated HA-R7BP-SS mutant. PC12 were transfected with HA-R7BP-SS and analyzed as indicated in the legend to B. These results are representative of three total such experiments performed with similar findings.

The Triton-insoluble pellet fraction contains aggregates of DRMs as well as non-raft associated detergent insoluble proteins [22]. To investigate whether the shift of endogenous of Gβ5/R7-RGS complexes to the detergent-insoluble fraction resulting from overexpression of wild type R7BP in PC12 cells reflects a shift of the complex to lipid rafts, the distribution of HA-R7BP and endogenous Gβ5 and RGS7 proteins along a sucrose density gradient was determined (Fig. 5). In control (vector-only) transfected PC12 cells a peak of endogenous Gβ5 and RGS7 was found in fractions 3 and 4 with the DRMs, while the vast majority of Gβ5/RGS7 immunoreactivity was found in fractions 6–10 among non-raft proteins (Fig. 5B), similar to the results in native PC12 cells (Fig. 1C). Approximately half of transfected wild-type R7BP migrates with the DRMs, and causes a major shift of endogenous Gβ5 and RGS7 proteins into fractions 1–5 as well (Fig. 5C). In contrast, transfected R7BP-SS was found in the heavier sucrose fractions with non-raft membrane protein markers and caused no shift of endogenous Gβ5 and RGS7 proteins into the DRM fractions (Fig. 5D). These results were entirely consistent with the cold Triton X-100 extraction data presented above and suggest the palmitoylation status of R7BP can govern the distribution of Gβ5/R7-RGS/R7BP complexes into or out of lipid raft membrane microdomains.

**Figure 6:**
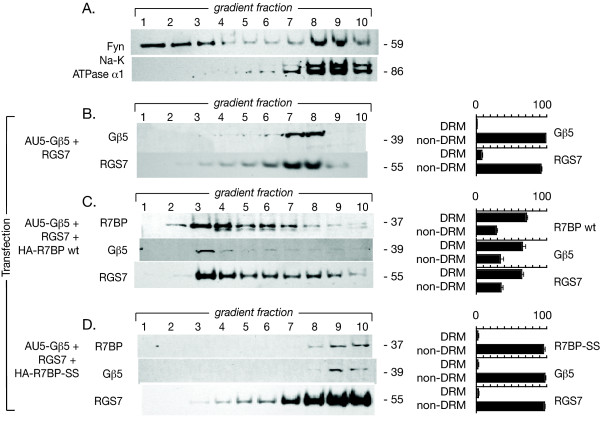
**Effect of R7BP on the distribution of the Gβ5/RGS7 complex among detergent-resistant membrane (DRM) and non-DRM fractions in HEK-293 cells**. A. Determination of endogenous DRM (lipid raft) and non-DRM markers along a sucrose density gradient in HEK-293 cells. The DRM (lipid rafts) were isolated using a sucrose density gradient prepared by ultracentrifugation. Fractions were analyzed by immunoblotting for Fyn as a marker for DRM and the α1 subunit of Na-K ATPase as a non-DRM marker. Fractions 1–5 were enriched in the DRM marker, while fractions 6–10 carried the non-DRM marker. B. Localization of transfected AU5-Gβ5 and RGS7 proteins in HEK-293 cells. Cells were transfected with the indicated constructs, lysed and subjected to a sucrose density gradient centrifugation. Equal aliquots from all ten fractions were subjected to SDS-PAGE followed by immunoblotting for the transfected Gβ5 and RGS7 proteins as indicated. Immunoblots of the sucrose density gradients shown were analyzed by densitometry and the distribution of the indicated immunoreactive bands between DRM and non-DRM fractions (as % of total immunoreactivity with that antibody) is shown as a histogram to the right of the corresponding immunoblot, with error bars representing the S.E.M. C. Localization of co-transfected wild type HA-R7BP, AU5-Gβ5 and RGS7 proteins in HEK-293 cells. Cells were co-transfected as indicted and analyzed as indicated in the legend to panel B. D. Localization of co-transfected HA-R7BP-SS, AU5-Gβ5 and RGS7 proteins in HEK-293 cells. Cells were co-transfected as indicated and analyzed as indicated in the legend to panel B. These results are representative of four total such experiments performed with similar findings.

Since neuron-like PC12 cells contain endogenous Gβ5 and RGS7 proteins [11,12], we wondered whether the effect of R7BP on lipid raft targeting could be verified in a non-neuronal cell line in which the components of Gβ5/R7-RGS/R7BP complexes were re-constituted by transfection. For this reason we analyzed the distribution of transfected R7BP, Gβ5 and RGS7 proteins in Triton X-100 extracts along a sucrose density gradient in HEK-293 cells (Fig. 6). HEK-293 cells were shown previously to lack significant expression of Gβ5 mRNA and protein [29]. As markers for DRM and non-DRM fractions in HEK-293 cells, the migration of the dually acylated kinase Fyn and the α1 subunit of Na-K ATPase, respectively, were determined by immunoblotting (Fig. 6A). Transfection of AU5 epitope-tagged Gβ5 and RGS7, without R7BP, resulted in no significant targeting of Gβ5/RGS7 complexes to the DRM fractions (Fig. 6B). On the other hand co-transfection of wild-type R7BP (Fig. 6C), but not the non-palmitoylated R7BP-SS mutant (Fig. 6D), with AU5-Gβ5 and RGS7 resulted in a shift of approximately 60% of the Gβ5/R7-RGS/R7BP complexes to the DRM fractions (Fig. 6C). When wild-type R7BP was employed, the peak of the low-density component of Gβ5/R7-RGS/R7BP complex proteins was in fractions 3 and 4 of the HEK-293 cell sucrose gradient (Fig. 6C), just as in PC12 cells. These findings show that, just as in neuronal PC12 cells that express the endogenous Gβ5 complex, in HEK-293 cells the palmitoylation status of R7BP can govern the distribution of Gβ5/R7-RGS/R7BP complexes into or out of lipid raft membrane microdomains.

### Endogenous mouse brain Gβ5, RGS7 and R7BP localize to lipid raft membrane domains

**Figure 7:**
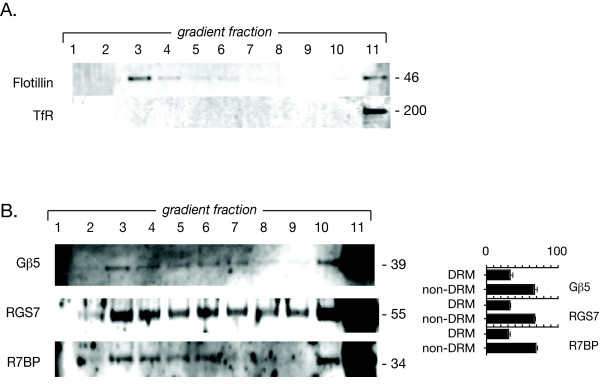
**Distribution of endogenous Gβ5/RGS7/R7BP among detergent-resistant membrane (DRM) and non-DRM fractions of mouse brain membranes**. A. Analysis of endogenous marker proteins in DRM and non-DRM fractions from mouse brain. Equal volumes of gradient fractions were analyzed by immunoblotting for flotillin-1, as marker for DRM, and the transferrin receptor (TfR) as a non-DRM marker. B. Sucrose density gradient analysis of endogenous Gβ5, RGS7, and R7BP synaptosomal membrane proteins in mouse brain. A detergent extract of mouse brain synaptosomes was subjected to a sucrose density gradient fractionation. Equal aliquots from all eleven fractions were subjected to SDS-PAGE followed by immunoblotting for the endogenous Gβ5, RGS7, and R7BP (using antibody TRS) proteins as indicated. Immunoblots of the sucrose density gradients shown were analyzed by densitometry and the distribution of the indicated immunoreactive bands between DRM and non-DRM fractions among (as % of total immunoreactivity with that antibody) is shown as a histogram to the right of the corresponding immunoblot, with error bars representing the S.E.M.

Since the fraction of endogenous Gβ5/R7-RGS protein complexes in neuron-like PC12 cells localized to the lipid rafts could be greatly increased by overexpression of wild-type R7BP, we investigated the localization of Gβ5/R7-RGS protein complexes in native brain where R7BP is most highly expressed [14,15]. Because of the high lipid and protein content of brain compared to cultured cells, a different sucrose density gradient protocol was utilized, resulting in 11 fractions per gradient [26,30]. After extraction of DRMs from a synaptosomal fraction of mouse brain, we found that a readily detectable fraction of endogenous brain Gβ5/RGS7/R7BP protein complexes localized to lipid raft fractions that comigrated with the flotillin marker (Fig. 7). As in neuronal PC12 cells, the bulk of brain Gβ5/RGS7/R7BP protein complexes distributed to the bottom gradient fractions where the non-raft protein transferrin receptor (TfR) marker is found (Fig. 7). In this sucrose density gradient protocol there is large component of incompletely solubilized synaptosomes that is represented in fraction 11, accounting for the heavy staining of marker and Gβ5/RGS7/R7BP proteins in this fraction.

## Discussion

It has been proposed that lipid rafts provide a concentrating platform for the efficient interaction of certain receptors and other signaling proteins [18,19,22]. The recently discovered Gβ5/R7-RGS anchoring protein R7BP, and its retinal paralog R9-anchoring protein, show structural similarity to the soluble N-ethylmaleimide-sensitive factor attachment protein receptor (SNARE) complex protein syntaxin [14,31]. SNARE complex proteins including syntaxin are highly enriched in lipid rafts [32]. Furthermore the dual palmitoylation of R7BP makes it a logical candidate for lipid raft targeting, given that the covalent fatty acylation of several signalling proteins with palmitate promotes their targeting to lipid rafts, including dually acylated heterotrimeric G protein α subunits [21]. The data presented here demonstrate that indeed the Gβ5/R7-RGS/R7BP complex associates with lipid rafts in native PC12 cells and mouse brain, and that targeting to these liquid-ordered membrane microdomains is supported by the wild-type, but not the non-palmitoylated mutant form of R7BP.

The present work shows that some 10–30% of endogenous Gβ5/R7-RGS/R7BP complexes are lipid raft-associated in native PC12 cells and brain. The data presented here suggest that R7BP and its palmitoylation status are the major determinants of the lipid raft association of Gβ5/R7-RGS/R7BP complexes. However, since a previous report found no significant association of RGS7 with lipid rafts in bovine brain membranes [13], it seems possible that additional signal(s) besides palmitoylation may be required to enhance the lipid raft targeting of the Gβ5/R7-RGS complex in native membranes. In photoreceptors for example the paralogous complex in the outer segments containing the retina-specific isoforms of Gβ5 and RGS9 undergoes a dramatic shift to the lipid raft membrane fraction only upon illumination, the external signal that initiates the phototransduction cascade [33]. By analogy with the retinal system, it may be that yet-to-be-identified external signal(s) regulate the concerted movement of neuronal Gβ5/R7-RGS/R7BP complexes to lipid rafts. The partitioning of these complexes to lipid rafts under basal conditions may be minimal.

The range of signal(s) that may control Gβ5/R7-RGS/R7BP complex subcellular localization and function is currently unknown. A recent study by Song and coworkers showed the importance of the R7BP C-terminal domain in the targeting Gβ5-RGS9-2 complexes to the plasma membrane and the nucleus, and demonstrated that a significant fraction of Gβ5/RGS9-2 is found in neuronal postsynaptic densities (PSD) [16]. The neuronal scaffolding protein PSD-95, though first identified and named for its association with PSD, is also highly enriched in rat brain synaptosomal lipid raft fractions [26], consistent with our results here showing that Gβ5/R7-RGS complexes comigrate with PSD-95 and LAT in the lipid raft fraction of PC12 extracts. The study by Song and coworkers did not address the localization of Gβ5/R7-RGS/R7BP complexes to lipid rafts [16]. Nevertheless the likelihood of functional connection between lipid rafts and PSD in the postsynaptic region of neurons makes the co-localization of Gβ5/R7-RGS/R7BP complexes to both lipid rafts and PSD a distinct possibility [26,27].

While the possible biological significance of Gβ5/R7-RGS/R7BP complex targeting to lipid rafts is presently unknown, strong clues can be assembled from currently available evidence. The most likely physiological target of Gβ5/R7-RGS/R7BP complex GTPase activating protein (GAP) activity, heterotrimeric Gαo [9,13,34], is acylated by both myristate and palmitate and is lipid raft-associated [21,28,35]. Recent studies in *Xenopus laevis *oocytes showed the regulatory effect of the Gβ5 complex on Gαi/o-coupled m2 muscarinic receptor signaling, typical of RGS protein-mediated Gαi/o-directed GAP action, was greatly enhanced by R7BP palmitoylation [15]. Furthermore the functional enhancement of R7BP due to palmitoylation in this pathway was a result of its membrane anchoring [17]. In light of these findings, our data suggest the targeting of the Gβ5/R7-RGS/R7BP complex to lipid rafts in neurons and brain, where G proteins and their effectors are concentrated, may be central to the G protein regulatory function of the complex.

## Conclusion

In both neuron-like PC12 cells and mouse brain, a fraction of endogenous Gβ5/R7-RGS/R7BP protein complexes is targeted to low-density, detergent-resistant membrane lipid rafts. In cultured PC12 and HEK-293 cells, the palmitoylation status of R7BP regulated the lipid raft targeting of endogenous or co-expressed Gβ5/R7-RGS proteins. Taken together with recent evidence that the kinetic effects of the Gβ5 complex on GPCR signaling are greatly enhanced by R7BP palmitoylation through a membrane-anchoring mechanism, our data suggest the targeting of the Gβ5/R7-RGS/R7BP complex to lipid rafts in neurons and brain, where G proteins and their effectors are concentrated, may be central to the G protein regulatory function of the complex.

## Methods

### Plasmid construction and antibodies

A cDNA encoding human R7BP was prepared from pCMV6-XL4 (GenBank XM_059682; Origene No. TC103542) by PCR subcloning of residues 2 to 257 into the BamHI (5') and EcoRI (3') sites of pcDNA3-HA vector thereby adding an in-frame N-terminal hemagglutinin (HA) 9-residue epitope tag (YPYDVPDYA). The C252S/C253S non-palmitoylated R7BP mutant (R7BP-SS), as described by Drenan and coworkers [15], was prepared using the QuickChange II Site-Directed Mutagenesis Kit (Stratagene) with the HA-tagged wild-type R7BP in pcDNA3 as the template. An AU5 epitope-tagged mouse Gβ5 cDNA was prepared by PCR subcloning of residues 2 -353 into the BamHI (5') and EcoRI (3') sites of pcDNA3-AU5 vector thereby adding an in-frame N-terminal AU5 6-residue epitope tag (TDFYLK). The expression construct encoding full-length bovine RGS7 in pcDNA4-HisMax-C (that adds an in-frame Xpress epitope and His-6 tag) was previously described [12]. The coding sequence of all constructs was confirmed by DNA sequencing. The primary antibodies used for immunoblotting were: mouse anti-HA monoclonal antibody (cat. No. MMS-101R, Covance), mouse anti-AU5 monoclonal antibody (cat. No. MMS-135R, Covance), affinity-purified rabbit ATDG polyclonal antibody against the N terminus of Gβ5 [10], purified IgG fraction of rabbit 7RC-1 polyclonal antibody anti-RGS7 [12], anti-R7BP N-terminal rabbit antibody (a generous gift from Dr. Kirill Martemyanov), anti-R7BP rabbit polyclonal antibody TRS (raised against the synthetic peptide CTRSSIFQISKPPLQSGDWERRG-amide (corresponding to residues 16 to 37 of human/mouse R7BP, with an added N-terminal cysteine) coupled to maleimide-activated KLH (Pierce), and affinity-purified on a column of synthetic peptide covalently bound to Ultralink Iodoacetyl Gel (Pierce)), mouse anti-flotillin-1 monoclonal antibody (cat. no. 610821, BD Transduction Laboratories), anti-LAT polyclonal rabbit IgG (cat. no. AB4093, Chemicon), anti-PSD-95 affinity-purified polyclonal rabbit antibody (cat. no. AB9634, Chemicon), anti-Na/K ATPase α1 subunit (cat. no. 06–520, Upstate Biotechnology), anti-Na/K ATPase β2 subunit (cat. no. A3979, Sigma), anti-transferrin receptor (TfR) (cat no. 13–6800, Zymed), mouse anti α-tubulin monoclonal antibody (cat. no. CP06, Calbiochem), and mouse anti-p84/N5 5E10 monoclonal (cat. no. MS-P8410-PX1, GeneTex). Secondary HRP-conjugated polyclonal antibodies employed were goat anti-mouse (cat. no. 1858413, Pierce Biotechnology) and goat anti-rabbit (cat. no. AP311, The Binding Site).

### Cell Culture

Rat pheochromocytoma PC12 cells (cells that synthesize dopamine and norepinephrine and express receptors for nerve growth factor, neuron-like features) were grown in 75 cm^2 ^flasks at 37°C and 5 % CO_2 _containing DMEM supplemented with 10 % horse serum, 5 % fetal bovine serum, 4 mM L-glutamine, 1 × penicillin/streptomycin (Biofluids, Rockville, MD) without nerve growth factor (supplemented DMEM). HEK-293 cells were grown under the same conditions as PC12 cells in DMEM supplemented with 10% fetal bovine serum, 4 mM L-glutamine and 1 × penicillin/streptomycin.

### Transient Expression of HA-R7BP (wild-type or -SS mutant) in PC12 cells, or RGS7 and Gβ5 without or with HA-R7BP (wild-type or -SS mutant) in HEK-293 cells

One day prior to transient transfection of HA-tagged wild-type or mutant R7BP, PC12 cells at 80 %-90 % confluence were plated in 75 cm^2 ^flask containing 15 ml supplemented DMEM without the addition of 1 × penicillin/streptomycin. After overnight incubation of the cells, 25 μg of plasmid DNA and 60 μl of Lipofectamine 2000 reagent (Invitrogen), diluted in 3 ml of Opti-MEM I medium (Gibco), were mixed, incubated at room temperature for 20 min, and added to each flask. The culture was incubated for 24 h in the same condition as mentioned above. Transfection of HEK-293 cells with RGS7 and Gβ5 without or with HA-tagged wild-type or mutant R7BP was performed using the same method. The expression of the transfected proteins was verified by immunoblotting and/or immunofluorescence.

### Gel Electrophoresis, immunoblotting and immunoprecipitation

Protein samples were separated on 4–20 % Tris-glycine gradient gels (Invitrogen) by SDS-polyacrylamide gel electrophoresis and transferred to a nitrocellulose membrane for 2 hr at 30 V in an XCell II Blot Module (Invitrogen). The membranes were blocked overnight in 5% milk in PBS containing 0.1% (v/v) Tween-20 (PBS/Tween). The next day the membranes were incubated with the primary antibody diluted into PBS/Tween for 30 min at room temperature. After rinsing in PBS/Tween, membranes were incubated with secondary antibody conjugated to HRP in PBS/Tween for 30 min at room temperature and rinsed again with PBS/Tween. The signal was detected using SuperSignal West Dura (cat. No. 34075, Pierce). Immunoprecipitation with anti HA antibody was performed according to the manufacturer's instructions (Immunoprecipitation Starter Pack, Cat. No. 309410, Amersham Biosciences). Briefly, after transfection and 24 h incubation, PC12 cells were lysed with buffer A (250 mM NaCl, 50 mM Tris, pH 8.0, 5 mM EDTA, 0.5 % NP-40 and 1 × complete protease inhibitor cocktail [cat. no. 1836170, Roche]). The lysate was centrifuged at 12,000 × g for 10 min at 4°C. The supernatant was incubated overnight with anti-HA antibody at 4°C. The mixture was then incubated with protein G-Sepharose beads previously equilibrated in buffer A for 2 hours with rocking at 4°C. The beads were then collected by centrifugation at 12,000 × g for 1 min, and washed 3 times with buffer A with harvesting of the beads by re-centrifugation, and the washes discarded. Proteins were eluted from the beads with SDS sample buffer and detected by immunoblotting.

### Immunofluorescence

PC12 cells were transfected as described above in chambers of poly-D-lysine-coated chamber slides (Nalge Nunc, No. 154941) and incubated for 24 h. The culture medium was discarded and the cells were rinsed with PBS and fixed in 4% formaldehyde for 30 min. The slides were rinsed with PBS and incubated with PBS containing 0.5% Triton X-100 for 5 min at room temperature. The slides were rinsed 3 times with PBS and blocked in blocking buffer (PBS containing 0.1% Triton X-100 and 1% BSA for 1 h at 37°C). Blocking buffer was discarded and the slides were incubated with the primary antibody diluted in blocking buffer for 1 h at 37°C. The slides were rinsed with washing buffer (PBS containing 0.1% Triton X-100) and incubated with the secondary antibody (Fluorescein isothiocyanate-conjugated donkey anti-mouse IgG, Jackson ImmunoResearch Labs, West Grove, PA) diluted in the blocking buffer for 1 h at 37°C. The slides were rinsed with washing buffer and Hoechst 33342 DNA-intercalating dye (0.5 μg/ml in PBS) was added to the cells in order to provide nuclear counterstaining. The cells were rinsed with PBS and one drop of mounting medium was added on the top of each slide. Finally the slides were covered with cover slide and confocal images were collected on a Leica SP2-UV 405 confocal microscope (Leica Microsystems, Exton, PA, USA).

### Cell Fractionation and Protein Determination

For separation of cytosol from a crude post-nuclear membrane fraction, PC12 cells were washed twice with cold PBS and harvested by centrifugation at 1,000 × g for 3 min at 4°C. Cell pellets were homogenized in a glass Dounce tissue grinder in hypotonic buffer consisting of 10 mM HEPES pH 7.5, 50 mM mannitol, 1 mM EDTA, 10 μg/ml soybean trypsin inhibitor, 0.5 μg/ml leupeptin, 2 μg/ml aprotonin and 1 μM pepstatin and then centrifuged at 1,000 × g for 3 min to remove unbroken cells and nuclei. The post-nuclear supernatant was separated into particulate and soluble fractions by centrifugation at 100,000 × *g *for 1 h as described [36]. For separation of cytosol and the nuclear fraction, the NE-PER kit (Pierce, No. 78833) was used according to the manufacturer's directions. The protein concentration in the different fractions was determined using the Bio-Rad protein assay kit with BSA as the standard.

### Triton X-100 solubilization experiments

Triton X-100 soluble and insoluble cell fractions were separated by the method of Fukata and coworkers [37]. The supernatant (Triton X-100 soluble) was separated from the pellet (Triton X-100 insoluble) by centrifugation at 20,000 × g for 10 min at 4°C. The supernatants were collected and the pellets were resuspended in SDS sample buffer in a volume equal to the volume of supernatant removed. Equal volumes of the supernatant and pellet fractions were then loaded in SDS-PAGE and proteins were analyzed by immunoblotting.

### Preparation of detergent-resistant membranes (DRMs) from PC12 and HEK-293 cells

DRMs were prepared by discontinuous sucrose density gradient centrifugation as described previously, with minor modifications [38,39]. Without transfection (for native cell membranes), or else after transfection and overnight incubation, 10^7 ^PC12 or HEK-293 cells were washed twice with ice-cold phosphate-buffered saline (PBS, pH 7.4) and harvested by centrifugation at 1,000 × g for 5 min at 4°C. Cell pellets were homogenized on ice in a glass Dounce tissue grinder in 0.4 ml of TE buffer (10 mM Tris-HCl, 4 mM EDTA) containing protease inhibitors. The mixture was centrifuged at 1,000 × g for 5 min at 4°C. 150 μl of the supernatant was incubated with 50 μl of TNE buffer (25 mM Tris-HCl, pH 7.4, 150 mM NaCl, 4 mM EDTA) containing 2 % of Triton X-100 (final Triton concentration of 0.5%) for 20 min on ice. 200 μl of the solution was mixed with 900 μl of TNE containing 85.5% of sucrose in Beckman SW55 tubes. The mixture was overlaid with 2.5 ml of TNE buffer containing 65% of sucrose and then with 1.1 ml of TNE buffer containing 10% of sucrose. The samples were centrifuged in a SW 55Ti rotor for 20 h at 170,000 × g at 4°C in a Beckman XL-90 Optima ultracentrifuge. Fractionation proceeded from the top of the gradient (0.47 ml each, for a total of 10 fractions). SDS sample buffer was added to equal aliquots of each fraction of the gradient and prepared for immunoblotting.

### Isolation of DRM from adult mouse brain

Lipid rafts were prepared from the synaptosomal fraction isolated from adult mouse brain according to published methods, with few modifications [26,30]. Fresh brain was isolated from an adult Black Swiss mouse and homogenized on ice in a glass Dounce tissue grinder in 16 ml of ice-cold solution A (0.32 M sucrose, 0.5 mM CaCl_2_, 1 mM each of NaHCO_3_, MgCl_2_, and orthovanadate, 20 mM of β-glycerophosphate and 5 μg/ml of leupeptin). After centrifugation at 700 × g for 5 min at 4°, the pellet was washed with solution A and re-centrifuged at 700 × g for 5 min. The supernatants were then combined and centrifuged as above. The resulting supernatant was centrifuged at 15,000 × g for 13 min and the pellet washed and centrifuged under the same conditions. The resulting pellet (P2), designated as crude synaptosomes, was suspended in 2.5 ml of ice-cold solution B (solution A without CaCl_2 _and MgCl_2_). After protein determination, proteins were solubilized by adding 2 ml of cold solution B containing 1% Triton X-100 per 3 mg protein of crude synaptosomes, followed by end-over-end mixing for 30 min at 4°C. The crude solubilizate was adjusted to 41% sucrose, and overlaid with 8 ml of 35% sucrose in solution B and 2.5 ml of 16% sucrose in solution B. DRM fractions were isolated by ultracentrifugation at 35,000 rpm, for 18 h, 4°C, using an SW41 rotor (Beckman Instruments Inc). Fractionation proceeded from the top of the gradient (1 ml each, for a total of 11 fractions). SDS sample buffer was added to equal aliquots of each fraction of the gradient and prepared for immunoblotting.

### Quantification of protein distribution between DRM and non-DRM fractions

The relative intensity of the major immunoreactive band(s) in each fraction of the sucrose density gradient was quantified by densitometric scanning using an Alpha Innotech Gel Imaging System and Alphamager™ 3400 software, as was an adjacent non-immunoreactive region of identical area (background). The net band density was obtained after subtraction of the background from the measured band density. The percentage of total band intensity represented by the DRM fractions and non-DRM fractions from cultured cells or mouse brain was determined by the sum of the density of the bands in fractions 1 to 5 and 6 to 10 respectively, divided by the total density which is the sum of the density of 10 fractions, multiplied by 100. Error bars in the histograms in the figures represent standard errors of the mean from multiple determinations.

## Abbreviations

G protein, heterotrimeric guanine nucleotide binding regulatory protein; RGS, regulator of G protein signaling; R7, the subfamily of RGS proteins including RGS6, RGS7, RGS9 and RGS11 in mammals; R7BP, R7-binding protein; GAP, GTPase activating protein; GGL domain, Gγ-like domain; NLS, nuclear localization signal; DRM, detergent-resistant membrane; LAT, linker for activation of T cells; PSD, postsynaptic density; TfR, transferrin receptor; S.E.M., standard error of the mean

## Competing interests

The author(s) declares that there are no competing interests.

## Authors' contributions

LN conceived the study with WFS and performed most of the experiments. AAW helped to set up and troubleshoot the sucrose density gradient experiments. MC performed the laser confocal microscopy experiments with LN. LMP and JHZ performed some experiments and provided critical analysis of the data. LN drafted the manuscript that was edited into final form by WFS. All authors have read and approved the final manuscript.

## References

[B1] Gilman AG (1995). Nobel Lecture. G proteins and regulation of adenylyl cyclase. Biosci Rep.

[B2] Watson AJ, Katz A, Simon MI (1994). A fifth member of the mammalian G-protein ^®^-subunit family. Expression in brain and activation of the ^®^2 isotype of phospholipase C. J Biol Chem.

[B3] Watson AJ, Aragay AM, Slepak VZ, Simon MI (1996). A novel form of the G protein beta subunit G^®^5 is specifically expressed in the vertebrate retina. J Biol Chem.

[B4] Cabrera JL, De Freitas F, Satpaev DK, Slepak VZ (1998). Identification of the Gβ5-RGS7 protein complex in the retina. Biochem Biophys Res Commun.

[B5] Snow BE, Krumins AM, Brothers GM, Lee SF, Wall MA, Chung S, Mangion J, Arya S, Gilman AG, Siderovski DP (1998). A G protein gamma subunit-like domain shared between RGS11 and other RGS proteins specifies binding to Gβ5 subunits. Proc Nat Acad Sci USA.

[B6] Snow BE, Betts L, Mangion J, Sondek J, Siderovski DP (1999). Fidelity of G protein β-subunit association by the G protein gamma-subunit-like domains of RGS6, RGS7, and RGS11. Proc Nat Acad Sci USA.

[B7] Levay K, Cabrera JL, Satpaev DK, Slepak VZ (1999). Gβ5 prevents the RGS7-Gao interaction through binding to a distinct Gγ-like domain found in RGS7 and other RGS proteins. Proc Nat Acad Sci USA.

[B8] Makino ER, Handy JW, Li TS, Arshavsky VY (1999). The GTPase activating factor for transducin in rod photoreceptors is the complex between RGS9 and type 5 G protein ^® ^subunit. Proc Nat Acad Sci USA.

[B9] Posner BA, Gilman AG, Harris BA (1999). Regulators of G protein signaling 6 and 7- Purification of complexes with Gβ5 and assessment of their effects on G protein-mediated signaling pathways. J Biol Chem.

[B10] Zhang JH, Simonds WF (2000). Copurification of brain G-protein β5 with RGS6 and RGS7. J Neurosci.

[B11] Zhang JH, Barr VA, Mo Y, Rojkova AM, Liu S, Simonds WF (2001). Nuclear localization of Gβ5 and regulator of G protein signalling 7 in neurons and brain. J Biol Chem.

[B12] Rojkova AM, Woodard GE, Huang TC, Combs CA, Zhang JH, Simonds WF (2003). Gγ subunit-selective G protein β5 mutant defines regulators of G protein signaling protein binding requirement for nuclear localization. J Biol Chem.

[B13] Rose JJ, Taylor JB, Shi J, Cockett MI, Jones PG, Hepler JR (2000). RGS7 is palmitoylated and exists as biochemically distinct forms. J Neurochem.

[B14] Martemyanov KA, Yoo PJ, Skiba NP, Arshavsky VY (2005). R7BP, a novel neuronal protein interacting with RGS proteins of the R7 family. J Biol Chem.

[B15] Drenan RM, Doupnik CA, Boyle MP, Muglia LJ, Huettner JE, Linder ME, Blumer KJ (2005). Palmitoylation regulates plasma membrane-nuclear shuttling of R7BP, a novel membrane anchor for the RGS7 family. J Cell Biol.

[B16] Song JH, Waataja JJ, Martemyanov KA (2006). Subcellular targeting of RGS9-2 is controlled by multiple molecular determinants on its membrane anchor, R7BP. J Biol Chem.

[B17] Drenan RM, Doupnik CA, Jayaraman M, Buchwalter AL, Kaltenbronn KM, Huettner JE, Linder ME, Blumer KJ (2006). R7BP augments the function of RGS7/Gβ5 complexes by a plasma membrane-targeting mechanism. J Biol Chem.

[B18] Resh MD (2006). Trafficking and signaling by fatty-acylated and prenylated proteins. Nat Chem Biol.

[B19] Brown DA (2006). Lipid rafts, detergent-resistant membranes, and raft targeting signals. Physiology (Bethesda, Md.

[B20] Prior IA, Hancock JF (2001). Compartmentalization of Ras proteins. J Cell Sci.

[B21] Moffett S, Brown DA, Linder ME (2000). Lipid-dependent targeting of G proteins into rafts. J Biol Chem.

[B22] Simons K, Toomre D (2000). Lipid rafts and signal transduction. Nat Rev Mol Cell Biol.

[B23] Webb Y, Hermida-Matsumoto L, Resh MD (2000). Inhibition of protein palmitoylation, raft localization, and T cell signaling by 2-bromopalmitate and polyunsaturated fatty acids. J Biol Chem.

[B24] Hering H, Lin CC, Sheng M (2003). Lipid rafts in the maintenance of synapses, dendritic spines, and surface AMPA receptor stability. J Neurosci.

[B25] Suzuki T, Ito J, Takagi H, Saitoh F, Nawa H, Shimizu H (2001). Biochemical evidence for localization of AMPA-type glutamate receptor subunits in the dendritic raft. Brain Res Mol Brain Res.

[B26] Besshoh S, Bawa D, Teves L, Wallace MC, Gurd JW (2005). Increased phosphorylation and redistribution of NMDA receptors between synaptic lipid rafts and post-synaptic densities following transient global ischemia in the rat brain. J Neurochem.

[B27] Suzuki T (2002). Lipid rafts at postsynaptic sites: distribution, function and linkage to postsynaptic density. Neurosci Res.

[B28] Arni S, Keilbaugh SA, Ostermeyer AG, Brown DA (1998). Association of GAP-43 with detergent-resistant membranes requires two palmitoylated cysteine residues. J Biol Chem.

[B29] Zhang JH, Lai ZN, Simonds WF (2000). Differential expression of the G protein β5 gene: Analysis of mouse brain, peripheral tissues, and cultured cell lines. J Neurochem.

[B30] Gil C, Cubi R, Blasi J, Aguilera J (2006). Synaptic proteins associate with a sub-set of lipid rafts when isolated from nerve endings at physiological temperature. Biochem Biophys Res Commun.

[B31] Hu G, Wensel TG (2004). Characterization of R9AP, a membrane anchor for the photoreceptor GTPase-accelerating protein, RGS9-1. Methods in enzymology.

[B32] Chamberlain LH, Burgoyne RD, Gould GW (2001). SNARE proteins are highly enriched in lipid rafts in PC12 cells: implications for the spatial control of exocytosis. Proc Natl Acad Sci USA.

[B33] Nair KS, Balasubramanian N, Slepak VZ (2002). Signal-dependent translocation of transducin, RGS9-1-Gβ5L complex, and arrestin to detergent-resistant membrane rafts in photoreceptors. Curr Biol.

[B34] Hooks SB, Waldo GL, Corbitt J, Bodor ET, Krumins AM, Harden TK (2003). RGS6, RGS7, RGS9, and RGS11 stimulate GTPase activity of Gi family G-proteins with differential selectivity and maximal activity. J Biol Chem.

[B35] Linder ME, Middleton P, Hepler JR, Taussig R, Gilman AG, Mumby SM (1993). Lipid modifications of G proteins: a subunits are palmitoylated. Proc Natl Acad Sci USA.

[B36] Jones TLZ, Simonds WF, Merendino JJ, Brann MR, Spiegel AM (1990). Myristoylation of an inhibitory GTP-binding protein alpha subunit is essential for its membrane attachment. Proc Natl Acad Sci USA.

[B37] Fukata M, Fukata Y, Adesnik H, Nicoll RA, Bredt DS (2004). Identification of PSD-95 palmitoylating enzymes. Neuron.

[B38] Waheed AA, Shimada Y, Heijnen HF, Nakamura M, Inomata M, Hayashi M, Iwashita S, Slot JW, Ohno-Iwashita Y (2001). Selective binding of perfringolysin O derivative to cholesterol-rich membrane microdomains (rafts). Proc Natl Acad Sci USA.

[B39] Ono A, Waheed AA, Joshi A, Freed EO (2005). Association of human immunodeficiency virus type 1 gag with membrane does not require highly basic sequences in the nucleocapsid: use of a novel Gag multimerization assay. J Virol.

